# Spatial characteristics and health risks assessments of trace metal pollution from road dusts in the industrialized city of Bangladesh

**DOI:** 10.1016/j.heliyon.2025.e42008

**Published:** 2025-01-16

**Authors:** Masum Howlader, Ashik Md Mamun, Mohammad Mahfuzur Rahman, Md Hasibur Rahman, Sadhon Chandra Swarnokar, Mahfuza Sultana, Md Tanvir Rahman, Tusar Kumar Das

**Affiliations:** a Department of Environmental Science and Technology, Jashore University of Science and Technology, Jashore, 7408, Bangladesh; b Department of Climate and Disaster Management, Jashore University of Science and Technology, Jashore, 7408, Bangladesh; c Environmental Science Discipline, Khulna University, Khulna, 9208, Bangladesh; d International Program in Hazardous Substance and Environmental Management, Chulalongkorn University, Bangkok, 10330, Thailand

**Keywords:** Street dust, Toxic trace metal, Pollution load, Spatial distribution, Human health risk

## Abstract

This study aimed to explore the presence of trace metallic pollutants and its associated health hazards in street dust from Gazipur City, one of Bangladesh's heavily industrialized urban centers. Thirty samples of street dust were collected, considering the city's industrial activity and population density. These samples underwent analysis using ICP-MS to detect potential toxic trace metals. Subsequently, the level of pollution and the associated human health risks were evaluated. The mean concentration of Bi (3.56 ± 2.10 mg/kg), Cd (0.71 ± 0.29 mg/kg), Co (4.03 ± 1.09 mg/kg), Cr (29.05 ± 25.44 mg/kg), Cu (17.36 ± 40.56 mg/kg), Ga (134.96 ± 45.39 mg/kg), In (2.25 ± 1.32 mg/kg), Mn (195.13 ± 191.15 mg/kg), Ni (20.31 ± 19.67 mg/kg), Pb (16.28 ± 3.68 mg/kg), Ti (1.72 ± 1.51 mg/kg) and Zn (44.96 ± 22.40 mg/kg). The element-specific environmental indices represent that the geo-accumulation index follows the descending order In (3.55 ± 1.54) > Bi (3.48 ± 1.08) > Ga (2.51 ± 0.48) > Cd (1.06 ± 0.73) > Pb (−0.23 ± 0.33), while the contamination factor fallows the descending order In (22.54 ± 12.57) > Bi (20.96 ± 12.13) > Ga (8.99 ± 2.97) > Cd (3.53 ± 1.41) > Pb (1.30 ± 0.29) > Zn (0.64 ± 0.31). The average calculated pollution load index is 0.68. The results of the dendrogram showed that those toxic trace metallic pollutants mainly originate from two different sources. The results of PCA (principal component analysis) also support the results of the dendrogram, which illustrate that the corrosion and weathering of automobile parts and other metal materials are the main contributors of Cd, Co, Cr, and Pb. In contrast, the use of leaded gasoline, corrosion in car surfaces and road paint are the main contributors of lead in road dust. The hazard index (HI) showed that Cr, Co, and Pb impose moderate to high non-carcinogenic human health risks. The infant's cancer risk for Cd (1.66^−05^) and Cr (1.59^−04^) is very close to the upper limit (10^−4^ to 10^−6^). Additional protective measures ought to be contemplated to diminish exposure to road dust among adults and infants.

## 1 Introduction

Road dust is defined as solid fine particles that come from a variety of emission sources, such as road traffic, domestic, and industrial emissions. These particles accumulate in urban environments and significantly contaminate them with trace metals [[1], [2], [3]] The pollution burdens in urban and suburban areas are increasing due to several factors: traffic emissions (from burning petrol, biomass, oil and coal), industrial emissions (from metallurgical production, chemical plants, and auto repair shops), power plant emissions (from burning diesel, coal, and furnace), and domestic emissions (from burning gas, biomass, oil, and coal) [[1], [2], [3], [4], [5], [6]]. Additionally, industrial effluents contribute to road dust accumulation, along with brake-wearing materials and tyre particles ejected from vehicles [2,7]. Road dust, which is prevalent in urban regions, is a composite of substances originating from a variety of sources including biogenic and anthropogenic [8,9]. Significant concentrations of Pb, Cd, Cr, Ni, Mn, Co, Cu, Zn, and Fe are present in these dust particles. Through atmospheric deposition mechanisms including sedimentation, impaction, and interception, trace metallic pollutants tend to accumulate in the surface soil [5,10]. Furthermore, mineral particles originating from soils near roadways are present in the dust [9,11].

Currently, the presence of small amounts of metallic contaminants in the environment has become a major cause for concern because of their harmful effects on both humans and the ecosystem. The presence of trace metals in road dust poses a significant concern as they can easily enter the human body through three main routes: ingestion, inhalation, and skin contact [12,13]. Exposure to these metallic pollutants can result in immediate health risks, such as disturbances in the endocrine system, the production of unstable molecules called free radicals and the displacement of vital components inside the body. Extended exposure to such substances can lead to a range of organic dysfunctions, dysplasia, and chronic diseases. This extended exposure can cause DNA damage and trigger mutagenic, teratogenic, and carcinogenic effects [14,15].

Presently, the swift and significant increase in urban environmental pollution is a pressing issue in emerging countries such as Bangladesh. The primary sources of air pollution in the country consist of gaseous emissions from vehicles, industrial chemicals, and conventional heating techniques. Bangladesh has a population growth rate of 2.2 % per year, which is one of the highest in Asia. This is accompanied by rapid urbanization. The combination of fast industry, urbanization, and population increase worsens environmental degradation [16,17]. In recent decades, the significant growth of human activities in Bangladesh has resulted in higher amounts of harmful metals being released into water, sediments, and soil [18,19]. The complex interactions of climate change and expanding urbanization have posed significant difficulties in accurately predicting the intricate patterns of long-range air movement of dust and short-term obstacles [2].

Prior research has extensively examined the levels, spatial distribution, and identification of sources of trace metals in road dust. Many of these studies have been conducted in developed countries [11,15,[20], [21], [22], [23], [24], [25], [26]]. Nevertheless, there is a lack of data on the concentration of trace metals in road dust specifically for poor nations. This information gap is evident in studies conducted by several research [[27], [28], [29], [30], [31], [32]]. Although Bangladesh has experienced substantial urban expansion and industrialization, there have been limited investigations into the toxicity burden of street dust. The studies conducted by Rahman et al. [2], and Rakib et al. [33], on the human-caused sources of trace metals in street dust from Dhaka and Narayanganj City respectively has offered valuable insights and called for additional research on larger scales [2,33]. Dhaka serves as the capital city, and the primary source of road dust is predominantly derived from household and roadway activity. Conversely, Narayanganj is an established industrial city situated on the banks of the Buriganga River, in close proximity to Dhaka. Thus, these two research offer insights from distinct viewpoints.

Gazipur is a neighboring city of Dhaka that has experienced substantial industrialization and economic growth in recent times. It is acknowledged as the central core of the textile industry in Bangladesh, hosting 75 % of the country's garment factories. It is designated as one of the 11 city corporations and acknowledged as a highly industrialized city in Bangladesh [34]. Gazipur is rapidly transforming into a densely populated industrial centre, with a population of almost 2.6 million residing in an area of approximately 329.53 km^2^. The proliferation of motor vehicles in Gazipur has been remarkable since the increasing prevalence of outdated technology vehicles has resulted in traffic congestion due to the diminishing availability of road space. Consequently, major traffic intersections have become focal points for the accumulation of air pollution resulting from car exhaust emissions. The city is experiencing both vertical and horizontal expansion without adequate consideration for spatial planning and environmental quality. It is crucial to offer scientific data on the current situation. Therefore, the aims of this investigation were threefold: i) to measure the levels of harmful trace metals in street dust in different areas of Gazipur City, ii) to map out how these trace metals are distributed throughout the city, and iii) to assess the potential health effects of trace metal contamination in street dust on both adults and children through different exposure routes. The findings of this study and the data from similar studies on older and different (diverse in nature) cities such as Dhaka and Narayanganj will provide valuable background information for developing policies that address public health, urban planning, and industrial zoning.

## 2 Materials and methods

### 2.1 Study area, sample collection, and analysis

**Fig. 1:**
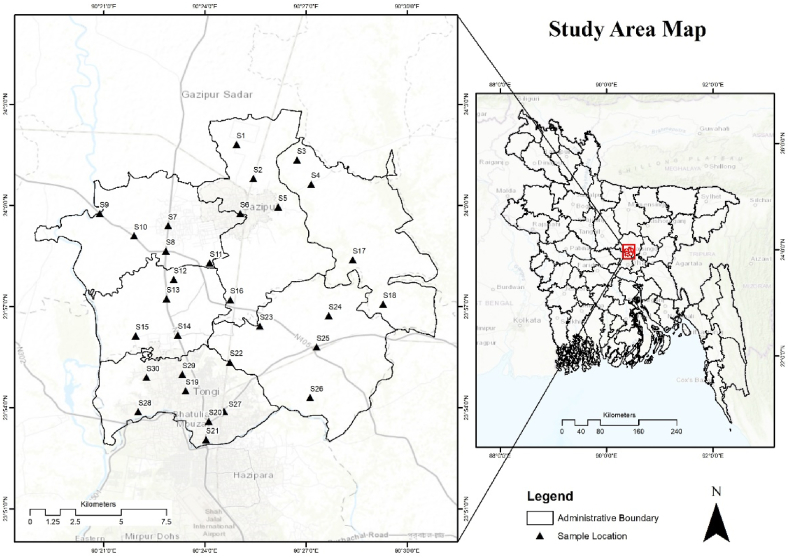
Location of sampling point along the study area.

The research was conducted in Gazipur city, situated north of the capital city of Dhaka, Bangladesh. Gazipur district lies in central Bangladesh, spanning between 23°53′–24°20′N and 90°9′–90°4′E, and contains a total area of 176,500 ha with a population of 123,531 individuals. Among the population, males comprise 52.52 %, while females make up 47.48 %. The population density stands at 2505 per square kilometer. The district experiences an annual average temperature ranging from a minimum of 12.7 °C to a maximum of 36 °C, with an annual precipitation of 2376 mm [34]. The total number of automobiles and industries in the area is rapidly increasing, with recent observations of smog in the Gazipur District indicating the initiation of air pollution [35]. Moreover, various industries such as metal smelting, textile, ceramic, glass, pharmaceutical, leather processing, and battery industries have been established in Gazipur District, Bangladesh (Fig. 1).

In December 2020, around 250 g of road dust were collected using a polyethene brush from impermeable surfaces. The dust samples were meticulously stored in airtight polyethene zip lock containers, properly marked, and subsequently transported to the laboratory. Upon arrival at the laboratory, all samples were subjected to a process of natural air drying for one week. Following this, the samples were passed through a 1.0 mm nylon mesh to separate any minute stones. Subsequently, the samples underwent oven-drying at a temperature of 85 °C for 24 h, after which they were passed through a mesh with a pore size of 74 μm.

One gram (1g) sample was digested at each sampling site using concentrated nitric acid (HNO_3_), concentrated perchloric acid (HClO_4_), and hydrogen peroxide (H_2_O_2_). The processed materials were further strained through Whatman-42 filter paper and transferred to 100 ml conical flasks. Analyzing duplicate samples, we used inductively coupled plasma optical emission spectrometry (ICP-OES, Spectro Genesis- FES27) to determine the concentration of trace metallic pollutants. These pollutants include aluminium (Al), cobalt (Co), lithium (Li), bismuth (Bi), nickel (Ni), cadmium (Cd), chromium (Cr), gallium (Ga), copper (Cu), indium (In), iron (Fe), lead (Pb), manganese (Mn), zinc (Zn), and titanium (Ti).

### 2.2 Data analysis

The data were subjected to statistical analysis using SPSS version 20. The spatial distribution of the metallic contaminants is evaluated utilizing ArcGIS version 10.5.

### 2.3 Geo-accumulation index (Igeo)

The *I*_geo_, also known as the Geo-accumulation Index, was developed by Muller in 1979 [36]. It is used to quantify the presence of trace metallic contaminants in dust samples. The *I*_geo_ was evaluated using the subsequent equation (1):

(1) $$\text{Geo}-\text{accumulation}\;\text{index}\;\left(I_{\text{geo}}\right)=\left(\log_{2}\frac{{Cm}_{Sample}}{1.5\times C_{ref}}\right)$$

In this index, *Cm*_*Sample*_ represents the trace metal content in the dust samples, while *C*_*ref*_ denotes the concentration of reference background soil. Additionally, a background matrix correction factor of 1.5 is applied to account for geochemical impacts. Muller's classification scheme for *I*_geo_ is outlined in Table 1.

**Table 1:** Categories of geo-accumulation index (Igeo).

*I*_geo_ class	*I*_geo_ o Value	*I*_geo_ value
0	Unpolluted	*I*_geo_ < 0
1	Unpolluted to moderately polluted	0 ≤ *I*_geo_ < 1
2	Moderately polluted	1 ≤ *I*_geo_ < 2
3	Moderately to strongly polluted	2 ≤ *I*_geo_ < 3
4	Strongly polluted	3 ≤ *I*_geo_ < 4
5	Strongly to very strongly polluted	4 ≤ *I*_geo_ < 5
6	Very strongly polluted	*I*_geo_ ≥ 5

### 2.4 Contamination factor

The metallic contamination of dust samples was assessed using the Contamination Factor (CF), proposed by Forstner and Wittmann [37]. CF was calculated using the following equation (2):

(2) $$\text{Contamination}\;\text{factor}\;\left(\text{CF}\right)=\left(\frac{{\text{Cm}}_{\text{Sample}}}{C_{ref}}\right)$$

In this calculation, *Cm* represents the quantity of the metal in the dust, while Cref denotes the concentration of the metal in reference background soil [37]. Detailed formulas for the calculation are provided in Table 2.

**Table 2:** Classification of contamination factor.

Pollution intensity	CF range
Low contamination	CF < 1
Moderate contamination	1 ≤ CF < 3
Considerable contamination	3 ≤ CF ≤ 6
High contamination	CF > 6

### 2.5 Pollution load index

The PLI (Pollution Load Index) of the street dust samples was determined using the following equation (3), as introduced by Tomilson, Wilson, Harris, and Jeffrey [38]:

(3) $$\text{PLI}={\left({\text{CF}}_{1}\times {\text{CF}}_{2}\times \ldots \times {\text{CF}}_{n}\right)}^{ˆ}\left(1/n\right)$$

Where: n is the total number of metals, and CF represents the quantity of each metal in the dust sample. Further details regarding the classification are provided in Table 3.

**Table 3:** Summary of the PLI classification.

Pollution intensity	PLI range
Denote perfection	PLI <1
Only baseline levels of pollutants are present	PLI = 1
Deterioration of site quality	PLI >1

### 2.6 Human exposure and health risk assessment

#### 2.6.1 Exposure assessment

The evaluation of human health risks related to trace metallic pollutants in street dust follows methodologies proposed by both the US Environmental Protection Agency [39] and the Dutch National Center for Public Health and Environmental Protection [40]. Considering the typical urban exposure patterns to trace metals present in road dust, three primary pathways may be identified: (a) direct ingestion (Ding); (b) inhalation (Dinh); and (c) dermal absorption (Ddermal) particles (Table 4). For assessing non-carcinogenic risks, the chronic daily dose (CDD: mg/kg/day) of highly toxic trace metals accepted via these exposure routes is determined using (4), (5), & (6) outlined below [41,42].

(4) $${\text{CDD}}_{\text{ing}}=C\times \frac{ingR\times EF\times ED}{BW\times AT}\times CF$$

(5) $${\text{CDD}}_{\text{inh}}=C\times \frac{inhR\times EF\times ED}{PEF\times BW\times AT\;}$$

(6) $$\text{CDDdermal}=C\times \frac{SAF\times SA\times DAF\times EF\times ED}{BW\times AT}\times CF$$

**Table 4:** Parameters used to measure chronic daily dose (CDD) by ingestion, inhalation exposure, and dermal contact for adult and children in health risk assessment calculations.

Factor	Definition	Unit	Adult	Children	Reference
C	Concentration of trace element in street dust	mg/kg	–	–	–
IngR	Ingestion rate	mg/day	100	200	USEPA (2002); Rahman et al., 2019; Chen et al., 2017
ED	Exposure Duration	years	30	6	
EF	Exposure Frequency	days/year	365	365	
BW	Average body weight	kg	70	15	
AT	Averaging time for non-carcinogenic	days	ED ×365	ED ×365	
CF	Conversion factor	kg/mg	$1\times 10^{-6}$	$1\times 10^{-6}$	
SA	Surface area of the skin in contact with dust	cm^2^	5700	2800	
SAF	Skin adherence factor for dust	mg/cm^2^/day	0.7	0.2	
InhR	Inhalation rate	m^3^/day	20	7.6	
PEF	Particle emission factor	m^3^/kg	$1.36\times 10^{9}$	$1.36\times 10^{9}$	
DAF	Dermal absorption factor (chemical specific)	unitless	0.001 for all elements except arsenic; 0.03(As)	0.001 for all elements except arsenic; 0.03(As)	US EPA (2004)

#### 2.6.2 Non-carcinogenic risk assessment

The HQ (Hazard Quotient) is commonly utilized to characterize non-carcinogenic risk, calculated as the ratio of the CDD (Chronic Daily Dose) to the RfD (Reference Dose) assigned for a specific substance [41]. Using the following equation (7):

(7) $$HQ=\frac{CDD}{RfD}$$

#### 2.6.3 Hazard index (HI)

The HI (Hazard Index) is a measure that accounts for the total contribution of doses from ingestion, dermal contact and inhalation. It is defined as Eq. (8):

(8) $$\text{HI}=\left(\frac{CDD}{RfD}\right)ingestion+\left(\frac{CDD}{RfD}\right)inhalation+\left(\frac{CDD}{RfD}\right)dermal$$

In this context.

• CDD represents the dose for element i, and RfD signifies the corresponding background dose for that specific element.
• The reference dose (RfD) and Slope Factor (SF) of metals are presented in Table 5 [43].

**Table 5:** Reference doses (RfD) for non-carcinogenic trace metals and slope factors (SF) for carcinogenic metals.

Heavy metals	Reference dose (RfD)			Slope Factor		
	Ingestion	Inhalation	Dermal	Ingestion	Inhalation	Dermal
(Cr)	3 ${\times 10}^{-3}$	${2.86\times 10}^{-3}$	${6\times 10}^{-5}$	${5.01\times 10}^{-1}$	$4.2{\times 10}^{1}$	${2\times 10}^{1}$
(Mn)	$4.6{\times 10}^{-2}$	${1.43\times 10}^{-5}$	${1.84\times 10}^{-3}$	–	–	–
(Pb)	3.5 ${\times 10}^{-3}$	${3.52\times 10}^{-3}$	${5.2\times 10}^{-4}$			
(Ni)	${2\times 10}^{-2}$	${2.06\times 10}^{-2}$	${5.4\times 10}^{-3}$		$8.4{\times 10}^{-1}$	
(Cu)	$4{\times 10}^{-2}$	$4.02{\times 10}^{-2}$	$1.2{\times 10}^{-2}$			
(Zn)	${3\times 10}^{-1}$	${3\times 10}^{-1}$	$6{\times 10}^{-2}$			
(Cd)	$1{\times 10}^{-3}$	$1{\times 10}^{-3}$	$1{\times 10}^{-5}$	–	$6.3{\times 10}^{0}$	
(As)	$3{\times 10}^{-4}$	$3.01{\times 10}^{-4}$	${1.23\times 10}^{-4}$	${1.5\times 10}^{0}$	${1.51\times 10}^{1}$	${3.66\times 10}^{0}$
• The ratio CDD/RfD is referred to as noncancer risk or HQ (Hazard Quotient).
• HI is utilized to assess the risk associated with elements across three exposure pathways: ingestion, dermal contact, and inhalation [41].

Risk categories are defined as follows.

• Negligible risk (Risk Category 1), where HI or HQ is < 0.1
• Low risk (Risk Category 2), where HI or HQ is ≥ 0.1 but <1.0
• Moderate risk (Risk Category 3), where HI or HQ is ≥ 1.0 but <4.0
• High risk (Risk Category 4), where HI or HQ is ≥ 4.0

#### 2.6.4 Carcinogenic risk assessment

Carcinogenic risk refers to the likelihood of developing cancer to an individual over their lifetime due to exposure to highly toxic contaminants [44,45]. The carcinogenic health risks associated with human exposure to toxic metals over a lifespan can be estimated using the following equations (9), (10) [41,42]:

(9) CR=CDD×SF

(10) TCR=∑CR

Here.

• CR represents the carcinogenic risk.
• TCR denotes the total carcinogenic risk.
• SF signifies the slope factor (mg/kg/day), as detailed in Table 5. According to USEPA [41], CR and TCR values below 1 × 10−61 × 10−6 are considered negligible, while values exceeding 1 × 10−41 × 10−4 are deemed detrimental to human health.

## 3 Results and discussion

### 3.1 General characteristics of trace metal pollution in street dust

Table 6 presents the extent of trace metal pollution in road dust within Gazipur city, along with background concentrations from various regions of Asian soil. The majority of the targeted metals investigated in the research exhibit concentrations higher than the reference level. Here, the average concentrations of Al and Fe were 5901.47 (±1619.74) and 7861.56 (±1233.58) respectively. The other studies in parts of the world also reported similar findings [46]. Al and Fe are noted as the most predominant elements in the environment [47], resembling the reference background concentration. The background concentration of Al and Fe in Bangladeshi soil is 26000 mg/kg and 28250 mg/kg respectively [48]. However, establishing natural background values of elements sourced from mobile sources like street dust in urban areas with significant anthropogenic activities remains challenging [46]. Additionally, the presence of huge electric vehicles, manual power transmission systems, and clutch wear could also contribute to increase Al levels in the study area [49].

**Table 6:** Statistics of trace metals in the road dust in the study area along with background concentration of Asian soil.

Element (mg/kg)	Min	Max	Mean	Median	SD	95%UCL	Background concentration	Reference
Al	3010.48	9474.24	5901.47	5979.53	1619.74	6506.29	26000 mg/kg	[48]
Bi	0.75	9.58	3.56	3.73	2.10	4.35	3.75 mg/kg	[49]
Cd	0.16	1.26	0.71	0.71	0.29	0.81	0.35 mg/kg	[64]
Co	2.19	7.61	4.03	3.96	1.09	4.44	13 mg/kg	[48]
Cr	7.29	93.69	29.05	20.06	25.44	38.55	114 mg/kg	[64]
Cu	1.34	228.53	17.36	8.13	40.56	32.50	32 mg/kg	[48]
Fe	5784.40	10504.26	7861.56	7762.39	1233.58	8322.19	28250 mg/kg	[48]
Ga	70.68	240.27	134.96	128.20	45.39	151.90	15.2 mg/kg	[52]
In	0.02	6.06	2.25	2.16	1.32	2.75	0.06 mg/kg	[52]
Li	1.57	6.31	3.75	3.87	1.18	4.19	–	–
Mn	95.55	1173.85	195.13	148.76	191.15	266.51	209 mg/kg	[64]
Ni	5.20	87.60	20.31	11.25	19.67	27.66	41 mg/kg	[48]
Pb	10.38	23.76	16.28	16.56	3.68	17.66	18 mg/kg	[48]
Ti	0.02	4.81	1.72	1.47	1.51	2.28	–	–
Zn	23.23	137.00	44.96	38.14	22.40	53.33	70 mg/kg	[48]

The average content of Bismuth (Bi) was 3.56 (±2.10) mg/Kg. Bismuth metal sulphides (Bi_2_S_3_) are frequently used in brake pad formulations to improve friction stability and reduce wear at high temperatures [50]. The average concentration of Bi in Indian soil is 3.75 mg/kg [49]. The soil generally contains a lithium (Li) level that is within the range of 1.57–6.31 mg/kg, with an average value of (3.75 ± 1.18) mg/kg. A study conducted in India reported a similar concentration of lithium in road dust [49]. Lithium-ion batteries are the main sources of lithium in the environment. They are commonly used in portable electronic gadgets and energy storage systems for electric cars [51]. The study area exhibits notable levels of gallium (Ga), with concentrations ranging from 70.68 to 240.27 mg/kg. Approximately 34 % of gallium is employed in optoelectronic devices, including laser diodes, photodetectors, light-emitting diodes, and solar cells. These devices are used in various industries such as consumer products, aircraft, medical equipment, industrial components, and telecommunication. High-performance computers, defense applications, and integrated circuits used in telecommunications account for the remaining 65 % of the demand for Ga [24]. The Ga concentration in all sample exceeds the background concentration (15.5 mg/kg) [52].

The investigated area shows a notable concentration of cobalt (Co), ranging from 2.19 to 7.16 mg/kg. Co is recognized as highly hazardous to both human health and the environment, primarily originating from vehicle emissions and industrial activities [49]. This hazardous metal having background concentration of 13 mg/kg, accumulates silently in the environment, often going unassessed in similar investigations. Comparable findings were noted for In (2.25 ± 1.32) and Ti (1.72 ± 1.51), indicating a greater abundance compared to the standard background level. The In concentration in most of the samples exceeds the background concentration of 0.06 mg/kg [52]. This raises concerns about the introduction of unfamiliar toxins into the ecosystem, with their sources largely unknown.

The Pb concentration in the present study was 16.28 (±3.28) mg/Kg, which is similar to the concentration observed in Dhaka city [2]. Some research reported elevated concentrations of Pb in dust samples in different parts of Bangladesh and India by Kabir (40.09 mg/kg) [53]; Ahmed and Ishiga (47.0 mg/kg) [5]; Suryawanshi (120.7 mg/kg) [54]; Ambade and Litrupa (534.6 mg/kg) [55], etc. The elevated presence of Pb in street dust can be attributed to various sources including past use and leakage of leaded gasoline, motor vehicle emissions, brick kilns, electric vehicles (battery-operated), industrial processes utilizing raw materials, commercial activities, combustion of fossil fuels, construction activities (coating usage, paint, and pigment), traffic congestion, wear of brakes, and loss of lead weights from wheels due to traffic [2,56,57]. However, some studies have reported lower concentrations of lead in street dust in various parts of the world, such as Victoria et al. with 5.5 mg/kg [58] and Rahman et al. with 18.9 mg/kg [2].

The copper (Cu) content in the current street dust sample is measured at 17.36 (±40.56) mg/kg, slightly below the prescribed value by CNEMC [59] and the majority of the sample exceeds the background concentration (32 mg/kg) of Bangladeshi soil [48]. Previous studies conducted in various parts of Bangladesh have reported slightly higher concentrations [2,5,53]. Literature indicates significantly higher concentrations of Cu observed in different regions worldwide [57,60,61]. The alarming increase in Cu pollution is attributed to various factors such as frequent and intensive application of gasoline-powered automobiles, heavy equipment workshops, brakes, mechanical abrasion of vehicles and car components, commercial activities, tire abrasion, congested traffic, presence of Cu in diesel and lubricants, industrial waste and effluents, car repair workshops, unregulated industrial and waste burning activities, as well as extensive automobile and industrial emissions [2,57,61,62].

The mean chromium concentration (Cr) in the dust sample is 29.05 (±25.44) mg/kg, surpassing the corresponding reference value provided by USEPA [63]. The average concentration of Cr in Indian soil is 114 mg/kg [64]. Comparatively, the concentration of Cr in Dhaka city is twice as high as observed in the present study [2,5,53]. The increased levels of Cr in street dust are predominantly attributed to discharged industrial waste and effluents. Major sources of Cr in urban street dust samples likely include vehicular traffic, car corrosion, combustion of fossil fuels, metal works, tannery and dyeing industries, power generation plants, application of anti-corrosion paints, and vehicle scrap workshops [2,65,66]. Additionally, research indicates that the production of automobile parts, stainless steel, titanium and aluminum alloy also contributes to the release of Cr into the environment [67].

The nickel (Ni) content in the dust samples ranges from 5.20 to 87.60 mg/kg, with the mean concentration closely aligning with the background levels [59]. The reference value of Ni in Bangladeshi soil is 41 mg/kg [48]. Several studies conducted in Dhaka city have reported slightly elevated Ni concentrations in dust samples [2,5,53]. The primary cause of Ni presence in urban street dust is the combustion of diesel fuel [60]. Additionally, the corrosion of car bodies and parts, municipal and industrial waste, accidental spillages of Ni-containing materials, and lithogenic factors (originating from local soil) could be the main Ni source in the urban environment [56,66].

The concentration of cadmium (Cd) in the study area ranges from 0.16 to 1.26 mg/kg. The reference value for Cd in soil is 0.11 mg/kg [59], with the highest observed value being 11 times greater than the reference. Elevated Cd concentrations may originate from metal processing, industrial activities, Cd-plated items, atmospheric emissions, etc. [68]. Additionally, Cd, Pb, and Zn are essential components of batteries, including those used in electric vehicles, which are becoming interestingly common in the Gazipur area. Improper collection of batteries or reprocessing leads to their disposal in the environment [2]. The observed Cd value along the study area is comparable to findings from other studies conducted in various parts of the world [56,69,70].

The zinc (Zn) concentration in the dust samples ranges from 23.23 to 137 mg/kg, with an average of 44.96 (±22.40) mg/kg. The highest concentration is double the reference background level 70 mg/kg [48]. In four sampling stations, the Zn concentration exceeds the background value, while it is lower in other stations. Zn concentrations may originate from tire wear, vehicle traffic, brake wear and including diesel exhaust emissions [57]. Several studies have reported that Zn pollution can result from vehicle repair workshops, heavy traffic, mechanical abrasion of vehicles, heavy equipment workshops, and metal smelting workshops [60,61].

A high concentration of manganese (Mn) is observed throughout the study area, ranging from 95.55 to 1173.85 mg/kg, with an average of 195.13 (±191.15) mg/kg. A similar Mn concentration was observed in southern India. Mn is a predominant component in street dust and is a frequent component in tire and brake wear [49]. It is crucial to assess the concentration of these metals in particulate matter suspended in the atmosphere. Bioavailability tests of these trace metallic pollutants can provide insight into the level of toxicity passed through various stages of the ecosystem and trophic levels.

### 3.2 Spatial distribution of metallic pollutants along Gazipur city

**Fig. 2:**
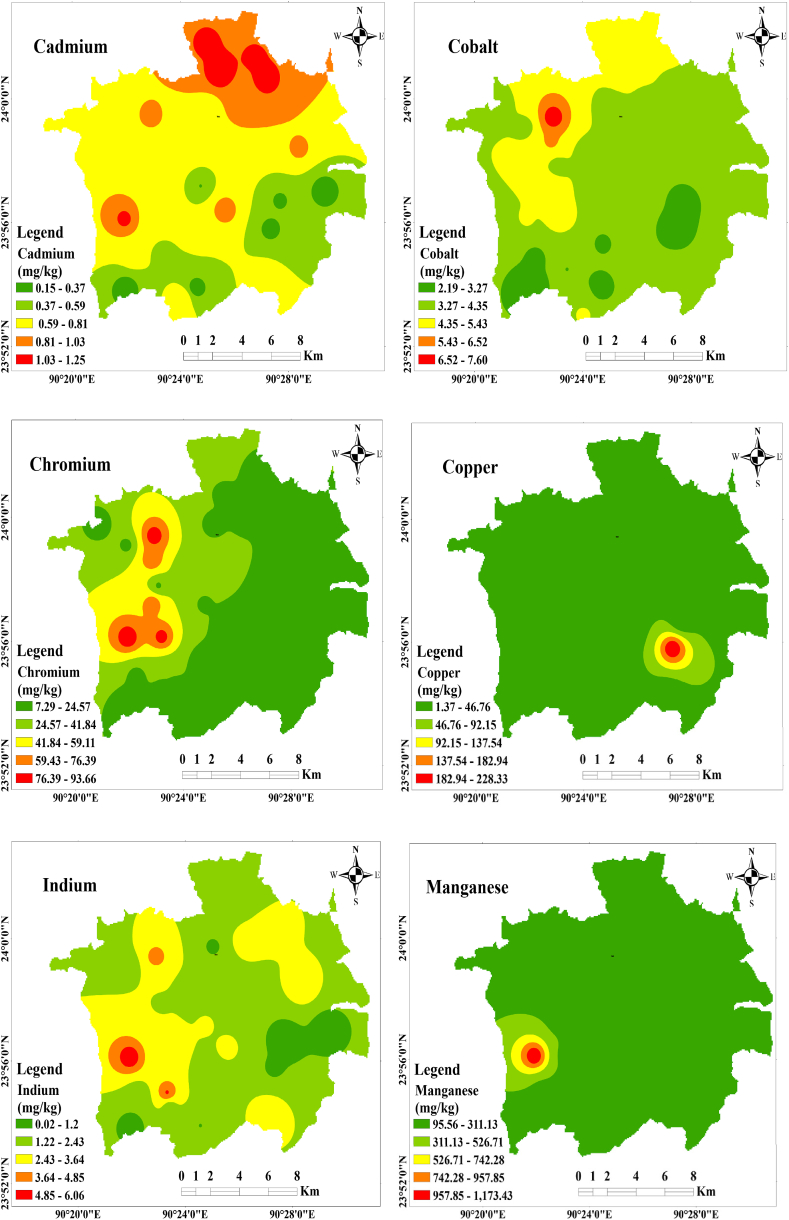

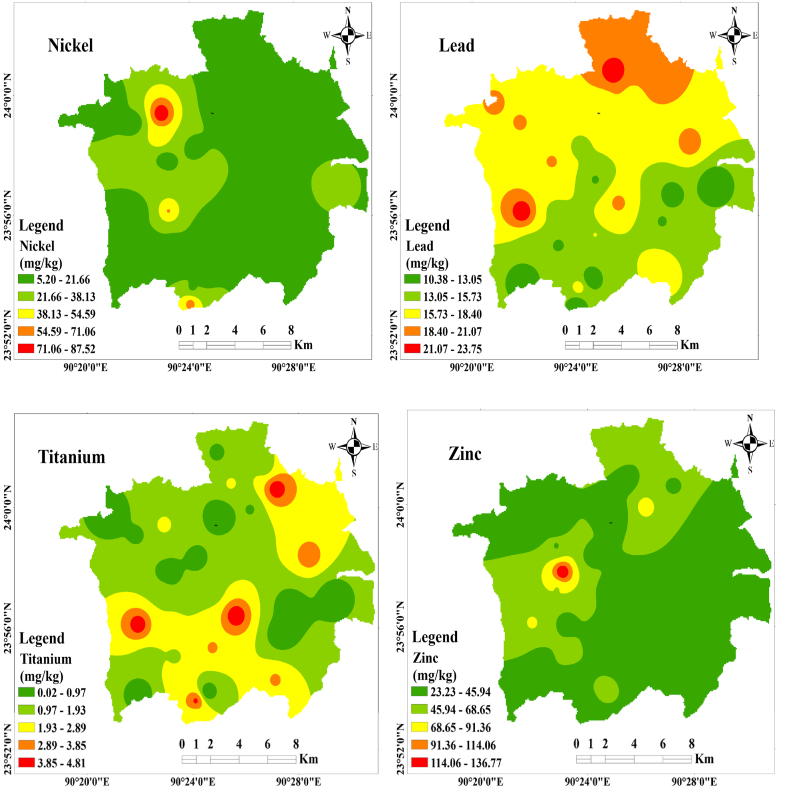
Spatial distribution of toxic trace metal along the study area.

Utilizing GIS applications enables the visualization of elemental-specific distributions across the study area and facilitates probable source detection [71]. Spatial distribution maps effectively portray the extent of potentially toxic metallic pollution throughout the study area. In this study, the Kriging interpolation method is used to estimate trace metal distribution, owing to the consistent elevation across the sampling locations. The spatial distribution of cadmium (Cd), cobalt (Co), chromium (Cr), copper (Cu), indium (In), manganese (Mn), nickel (Ni), lead (Pb), titanium (Ti), and zinc (Zn) in street dust samples across the study area is depicted in Fig. 2. The uniform terrain of the study area leads to varying concentrations of probable toxic metals in dust, influenced by factors such as traffic volume, population density, street configuration (e.g., junctions, main streets, bus stops), and land use patterns (industrial, residential, commercial zones).

Two distinct clusters of metallic pollution load are observed in the spatial distribution. The northern part of Gazipur City exhibits a high pollution load of Cd, Cr, Ni, and Pb, likely attributed to factors such as heavy traffic congestion, dense population, commercial activities, and extensive industrial operations in the area. The prevalence of pre-used leaded gasoline, industrial raw materials, electrical vehicle brick kilns, construction activities, and fossil fuel combustion from industrial exhausts may contribute to the accumulation of high concentrations of Pb in this region [2,51,71]. Additionally, the rapid industrialization in this area contributes to the high Cd concentration observed. The western zone demonstrates a second pollution-loaded area enriched with Cr, In, Mn, and Zn. The high chromium (Cr) concentration is linked to the presence of numerous garment dyeing industries, a large number of CNG-operated vehicles, congested traffic, and heavy vehicle activity in the western zone of Gazipur city. Elevated Zn and Ni concentrations in this area likely originate from metal smelting workshops, car repair workshops, heavy traffic, high population density, improper waste disposal, and car part manufacturing and abrasion [53]. Titanium (Ti), being a non-reactive material, exhibits high concentrations throughout Gazipur city, primarily stemming from noble industrial applications such as cosmetics, food, paint, and plastic industries [66]. Overall, the study highlights the accumulation of high concentrations of toxic trace metals in the study area due to anthropogenic activities.

### 3.3 Environmental pollution and ecological risks assessments

**Fig. 3:**
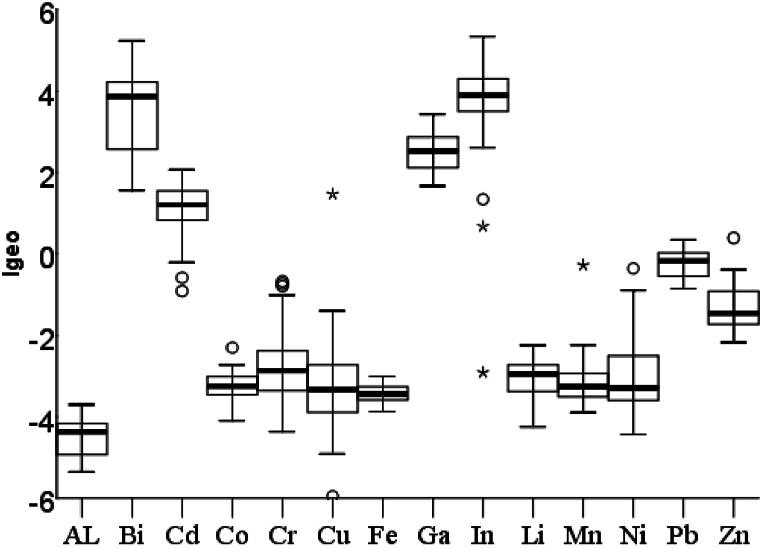
Geo-accumulation index of toxic trace pollutants along the study area.

The Igeo (Geo-accumulation index) served as a tool to evaluate the contamination levels of potentially toxic elements in street dust samples. The box plot of I_geo_ is represented in Fig. 3. The Igeo index follow the descending order of In (3.55 ± 1.54) > Bi (3.48 ± 1.08) > Ga (2.51 ± 0.48) > Cd (1.06 ± 0.73) > Pb (−0.23 ± 0.33) > Zn (−1.31 ± 0.45) > Cr (−2.71 ± 0.96) > Ni (2.87 ± 1.01). The average I_geo_ value represents that Al, Pb, Li, Cr, Cu, Fe, Mn, Co, and Zn were in an uncontaminated group while Cd was in the moderately polluted group. Gallium was moderately to strongly polluted. The interesting fact is that the relatively new metallic pollutants Bi and In fall in the strongly polluted category. These findings suggest a silent increase in new trace metal pollutants across the study area. It is crucial to investigate both trace and rare metal pollutants in this region.

The analyzed results show that three metallic pollutants show high contamination and it follows the descending order of In (22.54 ± 12.57) > Bi (20.96 ± 12.13) > Ga (8.99 ± 2.97). Cadmium shows considerable contamination (3.53 ± 1.41), while Pb shows moderate contamination (1.30 ± 0.28). The other metallic pollutants including Li, Al, Mn, Cr, Cu, Fe, Co, Ni, and Zn show low contamination (Table 7). The calculated mean PLI (pollution load index) value was 0.67 ± 0.15. All samples fall in the category of denote perfection except sample number 15 which falls in the category of deterioration of site quality. A PLI value exceeding 1 signifies a significant degradation of terrestrial environmental quality, attributed to both anthropogenic and geo-genic sources, alongside collective contributions from all measured elements [72]. The findings from the ecological risk assessment differ somewhat from typical studies. Several trace metal pollutants, which are not usually considered a threat, are disrupting the ecological balance more significantly than common pollutants.

**Table 7:** Contamination factors of metallic pollutants along the study area.

Pollution intensity	CF range	Metallic Pollutants
Low contamination	CF < 1	Al, Co, Cr, Cu, Fe, Li, Mn, Ni, Zn
Moderate contamination	1 ≤ CF < 3	Pb
Considerable contamination	3 ≤ CF ≤ 6	Cd
High contamination	CF > 6	Bi, Ga, In

### 3.4 Principal component analysis and hierarchical cluster analysis

Principal Component Analysis (PCA) methods were employed through SPSS software to discern the pollution sources of fifteen toxic metals. The Kaiser Meyer Olkin (KMO) Bartlett tests conducted on the logarithmic conversion of trace metal concentration data yielded a KMO value of 0.614, while the Bartlett tests indicated a significance level of 0.00, suggesting that the data were suitable for factor analysis and PCA [73]. The analyzed results are summarized in Table 8.

**Table 8:** Principal component analysis for source identification of trace metallic pollutants.

Variables	PC1	PC2	PC3	PC4
Al	**.900**	−0.326	0.066	−0.186
Bi	−0.507	0.438	**.675**	0.047
Cd	**.943**	−0.201	0.068	−0.092
Co	**.712**	0.418	−0.372	−0.351
Cr	0.521	**.726**	−0.280	0.065
Cu	−0.360	0.130	0.245	0.365
Fe	**.853**	0.220	0.054	0.201
Ga	**.768**	−0.383	−0.341	0.260
In	**.697**	0.522	0.302	0.181
Li	**.786**	−0.319	0.305	−0.310
Mn	0.473	0.418	0.246	0.486
Ni	0.051	**.777**	−0.392	−0.439
Pb	**.908**	−0.305	0.110	0.111
Ti	0.558	0.166	**.682**	−0.295
Zn	0.325	0.050	−0.283	**.704**
Eigenvalues	6.754	2.529	1.817	1.567
% of Variance	45.027	16.860	12.115	10.444
Cumulative %	45.027	61.887	74.002	84.446

Extraction Method: Principal Component Analysis.

Four principal components were extracted using the principal component analysis method, accounting for 84.446 % of the total variance. PC1 explained 45.027 % of the total variance and exhibited high positive loading factors on Al (0.9), Cd (0.943), Co (0.712), Fe (0.853), Ga (0.768), In (0.697), Li (0.786), and Pb (0.908) (Table 8). Pearson correlation coefficient analysis corroborated these findings, indicating a high correlation among these metals contributing to PC1. The contamination factor of Al and Fe in road sediment was not only similar but also high, suggesting their origin from natural sources (local soil). Traffic-related emissions are identified as the primary source of trace metals in road dust. Previous studies have highlighted automobile part corrosion and weathering, including coatings, guardrails, and paint, as major contributors to Cd, Co, Cr, and Pb in road dust [29]. The use of leaded gasoline, corrosion in-car surfaces, and road paint are attributed to increasing lead concentrations in road dust [72]. Similar findings from different studies indicate that Cu, Cd, and Pb in road sediment originate from traffic congestion, suggesting that PC1 is predominantly associated with transportation facilities and vehicle exhaust from anthropogenic activities.

PC2 accounted for 16.86 % of the total variance and showed high positive loadings for Cr (0.726) and Ni (0.777) (Table 8). The strong correlation between Cr and Ni suggests a common source, likely industrial discharge, traffic emissions, and paints. Studies conducted in the Dhaka city area also identified industrial activity as the primary source of Cr and Ni in road sediment [5], indicating that PC2 is closely associated with industrial discharge in the study area.

PC3, representing 12.11 % of the total variance, is predominantly characterized by Bi (0.675) and Ti (0.682), which showed no significant correlation with other metals, indicating a separate source of origin. PC4 accounted for 10.44 % of the total variance, with a high loading factor for Zn (0.704). The concentration of Zn is largely attributed to traffic volume, with studies suggesting that high Zn concentrations in road sediment arise from carburetors, car lubricants, and tire tread rubber [2].

**Fig. 4:**
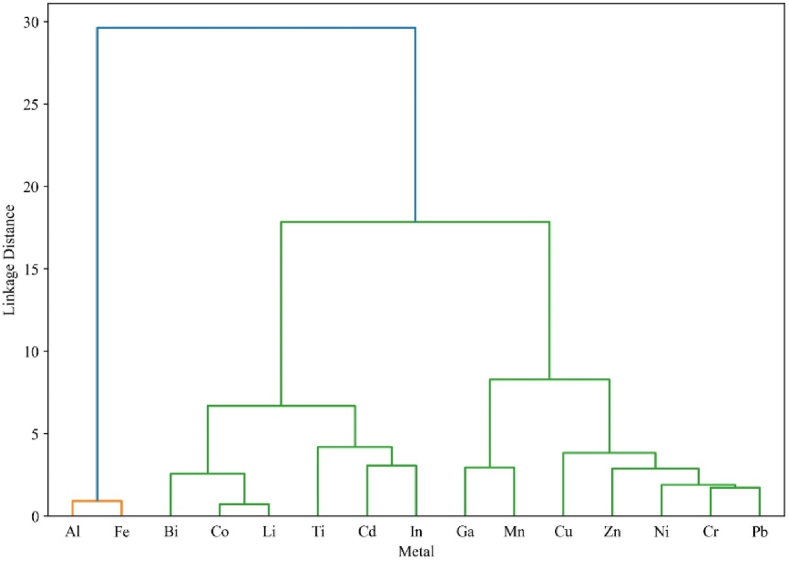
Dendrogram illustrating the hierarchical clustering analysis of trace metals recorded at various sampling locations.

Cluster analysis was employed to assess the similarities and differences among groups, resulting in three significant clusters. In this study, the cluster analysis findings are presented in Fig. 4. The first cluster indicates that the two predominant metals, Al and Fe, originate from similar sources. The second cluster includes Bi, Co, Li, Ti, Cd, and In, which share distinct characteristics of origin. These results suggest that many trace and rare metallic pollutants exhibit similarities not previously investigated in any other cities in Bangladesh. The third sub-cluster contains Ga, Mn, Cu, Zn, Cr, and Pb, most of which are trace metals and are linked to common sources.

### 3.5 Non-carcinogenic and carcinogenic risk assessment

The presence of trace metallic pollutants in street dust poses potential health risks to vulnerable populations with weakened immune systems [[74], [75], [76]]. These hazardous substances primarily penetrate into the human body by ingestion, followed by inhalation and dermal contact [66,77]. In the present study ingestion, inhalation, and dermal exposure pathways were considered to analyze non-carcinogenic risk (NCR) and carcinogenic risk (CR) of toxic metal in street dusts. Interestingly the chronic daily intake (CDI) in children is higher than the adults which point out that children are more sensitive to health hazards than adults due to exposure to contaminated street dust and several studies supported these results [66,78]. Hazard Index (HI) value was calculated to measure the non-carcinogenic health risks of ten trace metallic pollutants in children and adults (Table 9). Surprisingly, the HI value of all metallic pollutants indicates that non-carcinogenic risks will appear upon the residents if anthropogenic activities will continue to release pollutants into the environment. Three pollutants namely Co, Cr, and Fe already impose risks category 2 (low risks) along the study area. The HI value of all metallic pollutants follows the descending order of Cr > Co > Fe > Al > Mn > Pb > Cd > Ni > Cu > Zn.

**Table 9:** Non-carcinogenic and Carcinogenic health risk assessment for adults and children in Gazipur City.

Metals	HQ_ing_		HQ_inh_		HQ_dermal_		HI		Carcinogenic Risk		TCR	
	Adult	Child	Adult	Child	Adult	Child	Adult	Child	Adult	Child	Adult	Child
Al	8.42E-03	7.90E-02	8.67E-04	1.54E-03	3.36E-03	2.20E-03	1.26E-02	8.28E-02			4.78E-07	1.76E-04
Cd	2.01E-03	1.86E-02	1.48E-07	2.64E-07	4.02E-03	2.64E-03	6.04E-03	2.16E-02	1.66E-08	1.66E-05		
Co	1.92E-03	**1.79E-01**	1.41E-04	2.51E-04	–	–	2.05E-03	**1.81E-01**				
Cr	1.38E-02	**1.29E-01**	2.14E-06	3.79E-06	2.76E-02	**1.81E-01**	4.14E-02	**3.11E-01**	4.55E-07	1.59E-04		
Cu	6.19E-04	5.82E-03	9.07E-08	1.61E-07	8.24E-05	5.41E-05	7.02E-04	5.86E-03				
Fe	1.34E-03	1.25E-02	2.36E-05	4.19E-05	**2.03E-01**	**1.34E-01**	**2.05E-01**	**1.46E-01**				
Mn	6.05E-03	5.68E-02	2.86E-03	5.08E-03	6.05E-03	3.96E-03	1.49E-02	6.58E-02	6.36E-09	3.09E-07		
Ni	1.45E-03	1.36E-02	2.07E-07	3.68E-07	2.14E-04	1.40E-04	1.67E-03	1.37E-02				
Pb	9.64E-03	6.24E-02	9.72E-07	1.73E-06	1.78E-03	1.17E-03	8.43E-03	6.35E-02	5.16E-11	1.47E-08		
Zn	2.14E-04	2.01E-03	3.15E-08	5.59E-08	4.27E-05	2.81E-05	2.57E-03	2.03E-03				

Carcinogenic Risk (CR) refers to the likelihood of an individual developing any form of cancer due to prolonged exposure to pollutants throughout their lifetime [66,74]. Table 9 represents the carcinogenic risks of Cd, Cr, Mn, and Pb. The average carcinogenic risks of children are 1.76 × 10^−4^ indicates the strong possibility of cancer development among the children. Again, the cumulative carcinogenic risks of adults are 4.78 × 10^−7^ indicates carcinogenic risks of adults are negligible but it will it will detrimental to human health if anthropogenic pollution continues. Finally, it can be concluded that children will experience a higher cancer risk compared to adults because of trace metallic pollutants in street dust.

## 4 Conclusion

The current research investigates the contamination level of trace metallic pollutants in street dust, their spatial distribution along the study area and probable pollution source, and how those pollutants impose risks upon humans and the environment. The results showed that most trace metal pollutants have either exceeded or are approaching the reference background concentration, indicating their accumulation in street dust from various natural and anthropogenic sources. Surprisingly, a high concentration of several new metallic pollutants, such as Bi, Ga, In, and Ti, was observed in this area, indicating the emergence of new pollutants that were not previously considered a concern. Two pollution hotspots are identified in the northern and western parts of the study area, which are high-traffic induced, industrialized, and densely populated areas. Almost all metal pollutants are emitted due to high traffic volume, garments dying, metal works, corrosion of automobile parts, fuel, and industrial emissions. About half of the pollutants show high geo-accumulation along the study area while one-fourth show a high level of contamination. This is an alarming issue for the terrestrial environment. The hazard index shows that non-carcinogenic risks will appear if pollutants continue to be released into the environment. The Carcinogenic risk assessments show a strong possibility of cancer development among the children which is higher than the adults due to trace metallic pollutants in street dusts in Gazipur city. The main strength of this research is the quantification of several trace metallic pollutants, which has not been previously done in other studies. This offers valuable new insights for policymakers. However, a limitation of the study is the relatively low number of samples compared to the total area of Gazipur city. However, a comprehensive understanding of trace and emerging pollutant levels in industrialized cities of Bangladesh is essential for ensuring public health and environmental sustainability. The findings of this study will provide policymakers with insights into the situation regarding trace and emerging pollutants in densely populated urban areas of Bangladesh.

## CRediT authorship contribution statement

**Masum Howlader:** Methodology, Data curation. **Ashik Md Mamun:** Data curation. **Mohammad Mahfuzur Rahman:** Resources. **Md Hasibur Rahman:** Validation, Investigation. **Sadhon Chandra Swarnokar:** Formal analysis. **Mahfuza Sultana:** Writing – original draft. **Md Tanvir Rahman:** Investigation, Formal analysis. **Tusar Kumar Das:** Writing – review & editing, Supervision, Methodology, Conceptualization.

## Data availability statement

Data will be made available on request. For requesting data, please write to the corresponding author.

## Ethics declaration

The authors confirm that the research work is original and has not been published or submitted for publication elsewhere. All authors have unanimously approved the publication of the article. They also declare that if the article is accepted by *Heliyon*, it will not be published in any other journal or language without the written consent of the copyright holder.

## Declaration of competing interest

The authors declare that they have no known competing financial interests or personal relationships that could have appeared to influence the work reported in this paper.
